# Risk of heart disease in relation to radiotherapy and chemotherapy with anthracyclines among 19,464 breast cancer patients in Denmark, 1977–2005

**DOI:** 10.1016/j.radonc.2017.03.012

**Published:** 2017-05

**Authors:** Jens Christian Rehammar, Maj-Britt Jensen, Paul McGale, Ebbe Laugaard Lorenzen, Carolyn Taylor, Sarah C. Darby, Lars Videbæk, Zhe Wang, Marianne Ewertz

**Affiliations:** a Department of Oncology, Odense University Hospital, Denmark; b Institute of Clinical Research, University of Southern Denmark, Denmark; c Danish Breast Cancer Cooperative Group Secretariat, Copenhagen University Hospital, Rigshospitalet, Denmark; d Nuffield Department of Population Health, University of Oxford, United Kingdom; e Department of Cardiology, Odense University Hospital, Denmark; f Medical Research Council Population Health Research Unit, University of Oxford, United Kingdom

**Keywords:** Breast cancer, Heart disease, Radiotherapy, Anthracyclines

## Abstract

**Background and purpose:**

The risk of heart disease subsequent to breast cancer radiotherapy was examined with particular focus on women receiving anthracycline-containing chemotherapy.

**Material and methods:**

Women diagnosed with early-stage breast cancer in Denmark, 1977–2005, were identified from the register of the Danish Breast Cancer Cooperative Group, as was information on cancer-directed treatment. Information on heart disease was sought from the Danish National Patient and Cause of Death Registries. Incidence rate ratios were estimated comparing left-sided with right-sided cancer (IRR, LvR), stratified by calendar year, age, and time since breast cancer radiotherapy.

**Results:**

Among 19,464 women receiving radiotherapy, the IRR, LvR, was 1.11 (95% CI 1.03–1.20, *p* = 0.005) for all heart disease and among those also receiving anthracyclines the IRR, LvR, was 1.32 (95% CI 1.02–1.70, *p* = 0.03). This risk was highest if the treatment was given before the age of 50 years (IRR, LvR, 1.44, (95% CI 1.04–2.01) but there was no significant trend with age or time since treatment.

**Conclusions:**

Radiotherapy for left-sided breast cancer is associated with a higher risk of heart disease than for right-sided with the largest increases seen in women who also received anthracycline-containing chemotherapy.

The benefits of adjuvant radiotherapy for breast cancer have been demonstrated in randomized trials and include a substantial reduction in disease recurrence and a moderate reduction in breast cancer mortality after either breast conserving surgery or mastectomy with axillary dissection in axillary node-positive patients [1], [2]. However, in these trials the overall benefit from radiotherapy was reduced by an increased mortality from heart disease among women randomized to radiotherapy [3].

Women irradiated for left-sided breast cancer receive higher cardiac doses than women irradiated for right-sided breast cancer. In a previous study of almost 35,000 breast cancer patients from Denmark and Sweden for whom adjuvant radiotherapy was indicated, an increased incidence of all heart disease, and also of acute myocardial infarction, angina, pericarditis, and valvular heart disease was reported in women with left-sided breast cancer compared to women with right-sided breast cancer [4]. The Danish women in that study were diagnosed with breast cancer during 1977–2000 and followed to 2006. For these women, the predominant chemotherapy was cyclophosphamide, methotrexate and fluorouracil (CMF).

Anthracyclines, in the form of epirubicin, were first introduced into adjuvant chemotherapy for breast cancer in Denmark in 1990 as part of the DBCG 89D-trial [5]. Anthracyclines have been demonstrated to reduce breast cancer recurrence and mortality [6] but are cardiotoxic, increasing the risk of cardiomyopathy and congestive heart failure [7], [8]. Women with pre-existing heart disease, or who are at increased risk of heart disease, have a lower threshold for developing cardiotoxicity and are unlikely to receive adjuvant chemotherapy with anthracyclines. Therefore, the harmful effect of anthracyclines on the heart cannot be assessed by comparing women who did and did not receive it outside a randomized trial. However, it is possible to investigate whether the effect of radiotherapy on the heart is increased, when anthracyclines were also given, by comparing the incidence of heart disease in women with left-sided and right-sided breast cancer who received both radiotherapy and anthracyclines.

Few women diagnosed with breast cancer prior to 2000 received anthracyclines, so the previous cohort study provided little information regarding the effect of anthracyclines in addition to radiotherapy. We have therefore extended this Danish cohort to include breast cancer patients diagnosed up to the end of 2005 and followed for cardiac events until the end of 2013. In addition, we have obtained information on which women were actually given radiotherapy, rather than radiotherapy just being indicated, and also on which women were actually given chemotherapy and on the type of chemotherapy they received. The main aim of this study is to assess the risk of heart disease following breast cancer radiotherapy, especially among women who also received anthracycline-containing chemotherapy.

## Methods

### Material

Since 1977, the Danish Breast Cancer Cooperative Group (DBCG) has maintained a register of all women diagnosed with early-stage breast cancer in Denmark [9]. All Danish hospitals involved in the diagnosis and treatment of breast cancer patients report to the DBCG and its register is more than 95% complete. Women diagnosed with early-stage breast cancer from June 1977 to December 2005 were identified from the DBCG register. Women were excluded if they had bilateral cancer or cancer of unknown laterality, had a previous diagnosis of invasive cancer (apart from non-melanoma skin cancer), did not undergo surgery (e.g. had biopsy only), were diagnosed with breast cancer after emigration from Denmark or were aged less than 20 years or more than 80 years at diagnosis. Information on tumour characteristics and details of therapies actually received was also obtained from the DBCG register. Using the unique personal identification numbers issued to all Danish citizens, the DBCG register was linked with the Danish Register of Causes of Death (RCD) [10] for deaths since 1977, and the Danish National Patient Register (NPR) [11] for in-patient diagnoses since 1977 and outpatient diagnoses since 1995. Information was complete up to 31 December 2013. Information on prior cardiac disease, defined as ICD10-codes I00-I52 (or the corresponding ICD8-codes) at least 30 days before breast cancer diagnosis, was identified from the NPR.

The study was approved by the Danish Data Protection Agency and by the Danish Health and Medicines Authority.

### Statistical analysis

Descriptive analyses were conducted by tabulating the total number of women, and the number who received radiotherapy, according to breast cancer laterality and each of a number of other characteristics. For every value of each characteristic, a Mantel–Haenszel *X*^2^-test of whether the percentage given radiotherapy was the same for women with left-sided and right-sided cancer was conducted [12]. For further analyses, each woman’s contribution to the person-years at risk was calculated from six months after the date of breast cancer diagnosis and until the earliest of: diagnosis of any heart disease, 90th birthday, death, emigration, or 31 December 2013. For each endpoint of interest (all heart disease, ischemic heart disease, etc.) the total number of observed events and the person-years at risk were tabulated by calendar year of radiotherapy (starting from 1977–1980, then in 5-year groups), age at radiotherapy (starting from 20–24, then in 5-year groups) and time since radiotherapy (in 5-year groups). Tabulations were made for all women combined and for women in various different treatment categories. For women given radiotherapy, separate tabulations were made according to age at radiotherapy and years since radiotherapy. Incidence rate ratios comparing women in different categories, stratified by year, age, and time since breast cancer radiotherapy (all in 5-year groups), were estimated by logistic regression. To account for the differing numbers of women with left-sided and right-sided breast cancer, a variable taking the value of the ratio, left versus right, of the number of years at risk was included in the model, with the coefficient constrained to one. Tests of whether an individual rate ratio differed from one, of the homogeneity of the rate ratio across several different groups of women (for example those receiving different adjuvant medical treatments), and also tests for a trend in the rate ratio across several groups of women were based on the logarithms of the estimated rate ratios and their estimated variances. Calculations were performed using Stata version 13 [13]

## Results

**Fig. 1:**
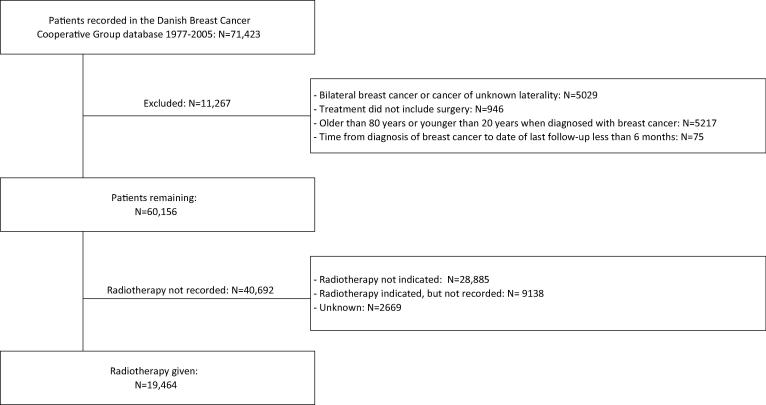
Identification of study population.

Between 1977 and 2005, 71,423 women were registered with a diagnosis of breast cancer by the DBCG. A total of 11,267 patients were excluded, leaving 60,156 women (Fig. 1). These women accrued 817,212 person-years, with a median follow up of 10.5 years (interquartile range 4.5–16.3 years).

When all 60,156 women were considered, the incidence rate ratio comparing women with left-sided breast cancer to women with right-sided breast cancer (IRR, LvR) for all heart disease was 1.05 (95% confidence interval (CI) 1.01–1.09, *p* = 0.01, Table 1). When the women were subdivided according to radiotherapy status, the IRR, LvR for the 19,464 for who received radiotherapy, was higher, at 1.11 (95% CI 1.03–1.20) and the significance level was more extreme (*p* = 0.005). In contrast, among women whose status was recorded in the DBCG as radiotherapy not indicated, radiotherapy indicated but not recorded, or radiotherapy unknown, the IRRs, LvR, were closer to unity and none were significantly increased.

**Table 1:** Incidence rate ratios, left-sided versus right-sided breast cancer, of all heart disease by laterality and radiotherapy status in 60,156 Danish patients, 1977–2005.

Radiotherapy status	Number of events/Number of women		Incidence rate ratio, left vs. right* (95% CI)	*p*-value†
	Left-sided	Right-sided		
Radiotherapy given	1482/9915	1291/9549	1.11 (1.03–1.20)	0.005
Radiotherapy not recorded	4586/21,076	4088/19,616	1.03 (0.99–1.08)	0.12
RT not indicated	3333/14,952	2949/13,933	1.04 (0.99–1.10)	0.10
RT indicated, but not recorded	835/4707	800/4431	1.00 (0.91–1.11)	0.93
Unknown	418/1417	339/1252	1.07 (0.92–1.24)	0.37
All women	6068/30,991	5379/29,165	1.05 (1.01–1.09)	0.01

Analysis based on the first diagnosis of heart disease occurring six months or more after the breast cancer diagnosis.

RT: radiotherapy, CI: confidence interval.

* Stratified by calendar year, age, and time since breast cancer radiotherapy.

† Test of whether incidence rate ratio is equal to unity.

Among the 19,464 women (32.4%) who received radiotherapy (Table 2) slightly fewer had left-sided than right-sided breast cancer (32.0% vs 32.7%) and, with such large numbers, this 0.7% difference was statistically significant (*p* = 0.05). When the analysis was repeated separately for women with different characteristics, differences of at least 1% in the proportion given radiotherapy for left-sided versus right-sided disease were seen for women who were: aged <50 years (1.0%), aged 70–79 years (1.1%), node positive (1.2%), given anthracyclines (1.9%), and for women given other types of chemotherapy (2.3%). However, as the numbers of women in the individual categories of these characteristics were smaller, the differences were, in most cases, not statistically significant. Among 4770 women who had prior heart disease recorded in the NPR, the percentage of women given radiotherapy was identical for women with left-sided and right-sided breast cancer. Among 3564 women receiving both radiotherapy and anthracyclines, chemotherapy dose was available for 94%, all receiving epirubicin. The median number of cycles was 7 (range 1–10), with a mean cumulative dose of 721 mg, corresponding to 410 mg/m^2^ of epirubicin. Anthracycline dose was unavailable for the remaining 6%, who received either epirubicin or doxorubicin.

**Table 2:** Characteristics of 19,464 Danish women given radiotherapy for early-stage breast cancer and of the total number of women diagnosed with the disease, by laterality of cancer.

Characteristic	Women given radiotherapy/total number of women (%)		*p*-value: % left vs. % right
	Left-sided	Right-sided	
*Year of breast cancer radiotherapy*			
1977–1989	1882/10,835 (17.4)	1834/10,009 (18.3)	0.07
1990–2000	3540/13,220 (26.8)	3279/12,397 (26.4)	0.55
2001–2005	4493/6936 (64.8)	4436/6759 (65.6)	0.29

*Age at breast cancer radiotherapy*			
<50	3352/8076 (41.5)	3344/7867 (42.5)	0.20
50–59	3185/8492 (37.5)	3088/8098 (38.1)	0.41
60–69	2666/8263 (32.3)	2409/7631 (31.6)	0.35
70–79	712/6160 (11.6)	708/5569 (12.7)	0.06

*Nodal status*			
Negative	3701/15,879 (23.3)	3469/14,823 (23.4)	0.84
Positive	6147/13,710 (44.8)	6032/13,122 (46.0)	0.06
Unknown	67/1402 (4.8)	48/1220 (3.9)	0.29

*Surgery*			
Mastectomy	4794/24,499 (19.6)	4581/22,854 (20.0)	0.19
Breast conserving surgery	5121/6492 (78.9)	4968/6311 (78.7)	0.82

*Adjuvant medical treatment*			
Anthracyclines	1777/2517 (70.6)	1787/2466 (72.5)	0.14
Other chemotherapy	1746/3832 (45.6)	1735/3620 (47.9)	0.04
Endocrine therapy (no chemotherapy)*	3191/6425 (49.7)	3034/6116 (49.6)	0.95
No adjuvant medical treatment recorded†	3201/18,217 (17.6)	2993/16,963 (17.6)	0.86

*Prior heart disease*++			
No	9230/28,543 (32.3)	8900/26,843 (33.2)	0.04
Yes	685/2448 (28.0)	649/2322 (28.0)	0.98

Total	9915/30,991 (32.0)	9549/29,165 (32.7)	0.05

* Included tamoxifen for 5 years to premenopausal women from 1999. For postmenopausal women, tamoxifen was given for 1 year from 1977 to 1995, and for 5 years from 1996. Aromatase inhibitors were introduced in a trial setting to postmenopausal women in 1998.

† Women recorded as having no adjuvant medical treatment (left: 2687/11,190, right: 2504/10,475); women who were allocated to a protocolled programme for systemic therapy but with no records on systemic therapy (left: 275/675, right: 263/664); and women not allocated to a protocolled programme (left: 239/6352, right: 226/5824).

++ Women recorded in the Danish National Patient Register as having heart disease prior to diagnosis of breast cancer.

When the 19,464 women who received radiotherapy were subdivided according to patient and tumour characteristics, there was no significant heterogeneity in the IRR, LvR according to year of breast cancer radiotherapy, age at breast cancer radiotherapy, nodal status, surgery, prior heart disease or adjuvant medical treatment given (*p* = 0.33) (Table 3). There were, however, some notable differences. For women given breast radiotherapy during 2001–2005 (i.e., the period in which anthracyclines were given) the IRR, LvR was significantly increased (IRR, LvR: 1.15, 95% CI 1.01–1.31), despite the fact that there was no increase in the previous decade (IRR, LvR: 1.01, 95% CI 0.90–1.14). The IRR, LvR was significantly increased for women receiving anthracyclines (IRR, LvR: 1.32, 95% CI 1.02–1.70, *p* = 0.03) and for women who did not receive systemic therapy (IRR, LvR: 1.15, 95% CI 1.02–1.31, *p* = 0.02). For women given chemotherapy other than anthracyclines the IRR, LvR, was 1.13 but this did not reach statistical significance. For women given endocrine therapy only, the IRR, LvR was 1.03 (95% CI 0.91–1.17).

**Table 3:** Incidence rate ratios, left-sided versus right-sided breast cancer for all heart disease by various characteristics in 19,464 Danish women given radiotherapy.

Characteristic	Number of events/number of women		Incidence ratio, left vs right* (95% CI)	*p*-value†	*p*-value for heterogeneity
	Left	Right			
*Year of breast cancer radiotherapy*					
1977–1989	402/1882	329/1834	1.24 (1.07–1.43)	0.004	0.09
1990–2000	561/3540	519/3279	1.01 (0.90–1.14)	0.86	
2001–2005	519/4493	443/4436	1.15 (1.01–1.31)	0.03	

*Age at breast cancer radiotherapy (years)*					
<50	311/3352	302/3344	1.04 (0.89–1.23)	0.57	0.53
50–59	432/3185	382/3088	1.14 (0.99–1.31)	0.07	
60–69	556/2666	434/2409	1.17 (1.03–1.32)	0.02	
70–79	183/712	173/708	1.00 (0.81–1.23)	1.00	

*Nodal status*					
Negative	623/3701	517/3469	1.14 (1.01–1.28)	0.03	0.73‡
Positive	849/6147	769/6032	1.11 (1.00–1.22)	0.04	
Unknown	10/67	5/48	1.66 (0.41–6.68)	0.48	

*Surgery*					
Mastectomy	701/4794	610/4581	1.13 (1.01–1.26)	0.03	0.73
Breast-conserving surgery	781/5121	681/4968	1.10 (0.99–1.22)	0.07	

*Adjuvant medical treatment*					
Anthracycline	140/1777	109/1787	1.32 (1.02–1.70)	0.03	0.33
Other chemotherapy	238/1746	215/1735	1.13 (0.94–1.37)	0.20	
Endocrine therapy (no chemotherapy)	548/3191	491/3034	1.03 (0.91–1.17)	0.59	
None reported	556/3201	476/2993	1.15 (1.02–1.31)	0.02	

*Prior heart disease*					
No	1255/9230	1084/8900	1.13 (1.04–1.22)	0.005	0.52
Yes	227/685	207/649	1.04 (0.86–1.28)	0.63	

All women given radiotherapy	1482/9915	1291/9549	1.11 (1.03–1.20)	0.005	

Analysis based on the first diagnosis of heart disease occurring six months or more after the breast cancer diagnosis.

* CI: Confidence Interval. Stratified by calendar year, age, and time since breast cancer radiotherapy.

† Test of whether incidence rate ratio is equal to unity.

‡ Excluding unknown category.

When all 19,464 women who received radiotherapy were considered, there were significant increases in the IRR, LvR, for ischaemic heart disease (1.18, 95% CI 1.03–1.35), angina (1.22, 95% CI 1.00–1.49) and valvular heart disease (1.39, 95% CI 1.00–1.96) (Table S1, panel a). These findings remained unchanged when the 18,130 women who did not have heart disease recorded in the Danish NPR prior to their cancer diagnosis were considered (Table S1, panel c). When 1134 women with heart disease recorded in the Danish NPR prior to their cancer diagnosis were considered, the IRR, LvR for all heart disease, was not significantly increased (1.04, 95% CI 0.86–1.28, Table S1, panel b). For myocardial infarction, however, the IRR, LvR was doubled (2.05, 95% CI 1.00–4.44). For all these three categories of women, the IRR, LvR, for heart failure was equal to or less than one (Table S1). Considering the 3564 women who were given both radiotherapy and anthracyclines, many types of heart disease contributed to the increased IRR, LvR (Table 4). For myocardial infarction, angina, and other and ill-defined heart disease the IRR, LvR was more than doubled but only for angina was the increase statistically significant when considered alone. For heart failure, the IRR, Lvs R was 1.51 (95% CI 0.64–3.56).

**Table 4:** Incidence rate ratios, left-sided vs right-sided, in 3564 Danish women diagnosed with breast cancer during 1990–2005 who were given both radiotherapy and anthracyclines, by type of heart disease.

Disease category (ICD-10 code)	Number of events Left/right	Incidence rate ratio, left vs. right* (95% CI)	*p*-value†
Ischaemic heart disease (I20-25)	32/19	1.71 (0.97–3.02)	0.07
Myocardial infarction (I21-23, I252)	7/3	2.29 (0.59–8.90)	0.23
Angina (I20)	21/9	2.36 (1.08–5.17)	0.03
Other ischaemic heart disease	4/7	0.58 (0.17–2.01)	0.39

Other heart disease (I00-52 excluding I20-25)	108/90	1.23 (0.93–1.64)	0.15
Hypertensive heart disease (I10-15)	14/21	0.59 (0.30–1.18)	0.14
Pulmonary embolism (I26-28)	11/8	1.33 (0.52–3.39)	0.55
Pericarditis (I01.0, I09.2, I30-32)	14/8	1.67 (0.69–4.03)	0.28
Valvular heart disease (I00-09, I01.0, I09.2, I34-39)‡	8/5	1.96 (0.59–6.52)	0.27
Other rheumatic heart disease (I00.9)	0/1	-	-
Acute endocarditis (I33)	1/1	0.99 (0.06–15.9)	1.00
Myocardial disease (I40-43)	11/9	1.26 (0.52–3.03)	0.61
Conduction disorders & arrhythmias (I44-45, I47-49)	32/26	1.32 (0.78–2.22)	0.30
Cardiac arrest (I46)	1/1	1.06 (0.07–16.9)	0.97
Heart failure (I50)	13/9	1.51 (0.64–3.56)	0.34
Other & ill-defined heart disease (I51, I52)	3/1	2.68 (0.30–27.6)	0.36

All heart disease (I00-52)	140/109	1.32 (1.02–1.70)	0.03

1777 women had left-sided breast cancer and 1787 women had right-sided breast cancer. Analysis based on first diagnosis of heart disease six months or more after breast cancer diagnosis.

Results for other categories of women are given in Table S1.

* CI: Confidence Interval. Stratified by calendar year, age, and time since breast cancer radiotherapy.

† Test of whether incidence rate ratio, left vs right, is equal to unity.

‡ Left-sided: aortic 1, mitral 5, pulmonary 2. Right-sided: aortic 2, mitral 3.

**Fig. 2:**
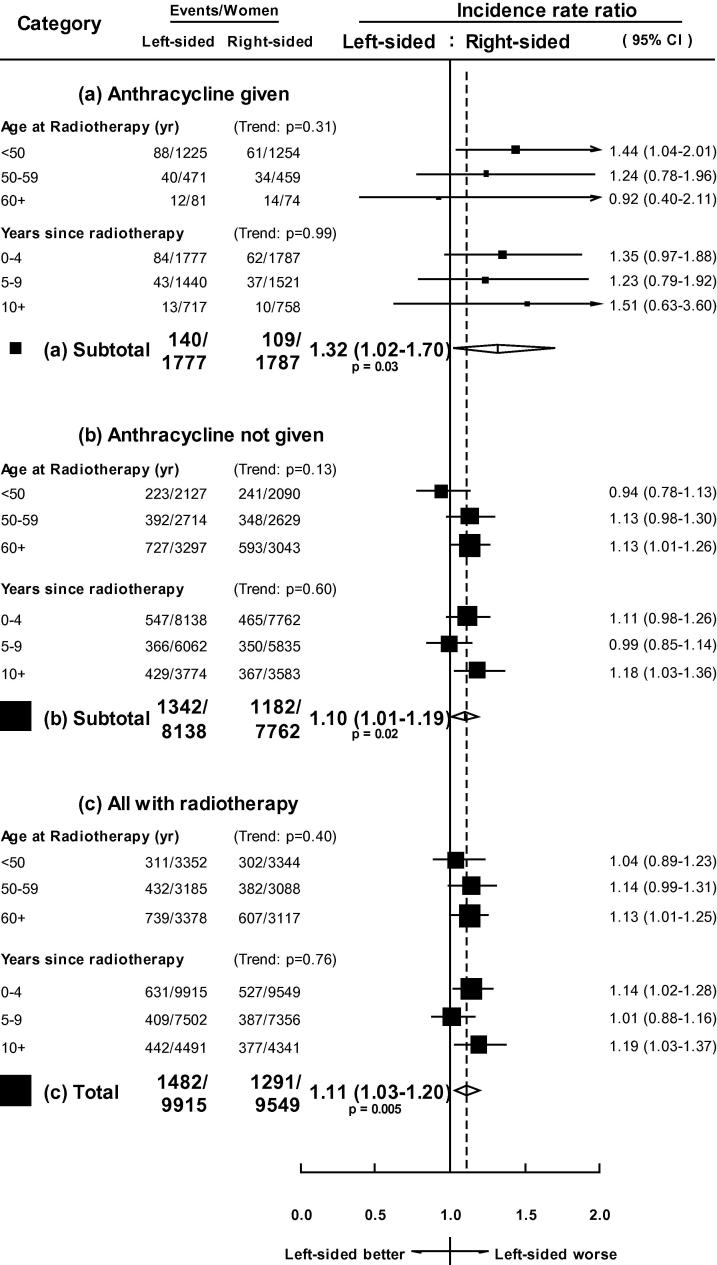
Incidence rate ratios, left-sided versus right-sided breast cancer, of all heart disease in 19,464 women given radiotherapy by age at radiotherapy, years since radiotherapy, and whether or not anthracycline chemotherapy was given.

When women given both radiotherapy and anthracyclines were subdivided according to the age at which radiotherapy was given, the highest IRR, LvR was for women aged less than 50 years when treated (IRR, LvR: 1.44, 95% CI 1.04–2.01, Fig. 2, panel a). However, there was no significant trend in the IRR, LvR across the three age-groups <50, 50–59, and 60+ years either for women who received anthracyclines, or for all other women given radiotherapy (Fig. 2 panel b), or for all women given radiotherapy (Fig. 2 panel c). The trend in the IRR, LvR, across time-periods 0–4, 5–9, and 10+ years after radiotherapy, was not significant for women given anthracyclines and radiotherapy, for other women given radiotherapy, or for all women given radiotherapy.

We examined the mortality rate ratios, left-sided vs right-sided, by cause of death (Table S2). Only 12 women (3 left-sided and 9 right-sided) died of heart disease among those receiving both radiotherapy and anthracyclines. The majority were registered as having died from breast cancer which was the main contribution to increased all-cause mortality ratio, left-sided versus right-sided of 1.26 (95% CI 1.10–1.44, *p* < 0.001). All-cause mortality did not differ significantly between left- and right-sided breast cancers among women receiving radiotherapy but not anthracyclines.

## Discussion

This study confirms previous observations that women who received radiotherapy for left-sided breast cancer had an increased risk of subsequent heart disease compared with women who received right-sided radiotherapy. The present study has examined the radiation-related increase in heart disease according to the type of systemic therapy given. It found that the proportional increase among women irradiated for left-sided cancer compared with right-sided cancer was highest for women who also received chemotherapy with anthracyclines. Among women given both anthracyclines and radiotherapy, the risk of heart disease was higher in left-sided breast cancer compared with right-sided, even though most of them had been treated since the year 2000 using modern radiotherapy techniques. This suggests that chemotherapy with anthracyclines may increase the effect of radiotherapy on the heart.

Initial exploratory analyses compared women who received adjuvant therapy with those who did not and showed that women who received adjuvant therapy had lower heart disease rates than those who did not. This suggests that women who were at higher risk of heart disease were less likely to receive radiotherapy and adjuvant medical treatment, making comparisons of women who did and did not receive such therapies biased. We have demonstrated previously [4] that radiotherapy was associated with reduced mortality from heart disease in women given breast-conserving surgery and increased mortality among other women, leading to selection bias if comparisons are made between irradiated and unirradiated women. Such comparisons should not be used to draw reliable conclusions as to the presence of any radiation-related risk.

More than thirty studies, including around a quarter of a million women irradiated for breast cancer during the past five decades, have reported heart disease rates in women irradiated for left-sided cancer compared with women irradiated for right-sided cancer [14]. In most of these studies, the left versus right cardiac event rate ratio was greater than one and, in studies where specific types of heart disease were examined, ischaemic heart disease was the type most commonly affected. Consistent with these, in the present study, among all women receiving radiotherapy during 1977–2005, the increase in heart disease among women irradiated for left-sided breast cancer compared with right-sided cancer there was a clear increase in ischaemic heart disease.

Anthracyclines were introduced into adjuvant chemotherapy as part of a randomized trial for breast cancer patients in Denmark in 1990. It improves both breast cancer and overall survival [5], [6]. However, several studies have shown that it can cause heart failure, with the risk increasing according to cumulative anthracycline dose [7], [8]. Most studies of radiation-related heart disease have not included women who also received anthracyclines. The relationship between heart radiation dose and subsequent ischaemic heart disease has been studied in a population-based case-control study in which individual cardiac doses were estimated. The major coronary event rate increased approximately linearly with mean heart radiation dose by 7.4% per Gray (95% CI, 2.9 to 14.5; *p* < 0.001) [15]. This study included very few women who also received anthracyclines. Information is therefore needed on whether the slope of this dose response relationship is affected by exposure to anthracyclines and on whether breast cancer radiotherapy given in combination with anthracyclines increase the risk of heart failure.

### Strengths and limitations of the study

A strength of our study is that it is based on the entire population of Danish breast cancer patients treated between 1977 and 2005. We included only women for whom there was a record that radiotherapy had actually been given, rather than just being indicated, as in previous studies from the DBCG. This will have removed the downward bias in the IRR, LvR previously caused by including women who were not, in fact, given radiotherapy. We also were able to classify women according to the type of chemotherapy given, enabling analyses of the cardiotoxic effects of radiotherapy and anthracyclines combined. Even with these stringent criteria, our study included nearly 20,000 women who received radiotherapy, 3500 of whom also received anthracyclines.

The comparison of cardiac events in women irradiated for left-sided versus right-sided breast cancer represents a comparison of higher versus lower radiation dose. Up until 2002 radiotherapy regimens in Denmark were identical in women with left-sided and right-sided breast cancer. During 2003–2005 some women were entered into the DBCG-IMN study in which internal mammary node radiation was given to women with right-sided but not with left-sided breast cancer [16]. This may be one of the reasons for the increased breast cancer and all-cause mortality rates among women with left-sided cancer who received both radiotherapy and anthracyclines (Table S2). Even for the women in this study, however, cardiac doses were higher in women with left-sided than right-sided breast cancer. In most countries, the ratio of dose in left-sided versus right-sided breast cancer varies substantially according to the regions irradiated [17]. We did not have heart dose information for the individual women in our study. Nevertheless, a study of heart doses from Danish breast cancer regimens used during 1977–2001 has shown that the average mean heart dose was around 6 Gy for left-sided RT and around 2–3 Gy for right-sided radiotherapy [18] though with considerable variation from 1 Gy to 8 Gy for left-sided tangential radiotherapy [19].

In this study, the number of women receiving both radiotherapy and anthracyclines was limited and the length of follow-up was relatively short which is clearly a limitation of our study. In addition, women selected for chemotherapy with anthracyclines are less likely to suffer from heart disease at diagnosis. The study also has some further limitations. First, the cardiology records of patients recorded with heart disease were not examined to confirm diagnoses from the Danish NPR. Second, for angina it is possible that women who developed chest pain after receiving left-sided radiotherapy may have been more frequently investigated, and diagnosed with angina than women receiving right-sided radiotherapy. This may have affected our IRR, LvR ratios for angina. Third, we were not able to examine the effect of trastuzumab because no women in our study received it.

### Implications

Our results have implications for assessment of the risk of today’s breast cancer radiotherapy. They confirm the excess of ischaemic heart disease from breast cancer radiotherapy seen in previous studies and suggest that the effect of radiotherapy on the heart may be increased by anthracyclines. From a clinical point of view, every possible effort should be made to reduce the dose to the heart in breast cancer radiotherapy but without compromising the coverage of the target, thus maintaining the beneficial effect of radiotherapy.

## Conclusion

Radiotherapy for left-sided breast cancer is associated with a higher risk of heart disease than for right-sided with the largest increases seen in women who also received anthracycline-containing chemotherapy.

## Funding

Oxford funding was provided by Cancer Research UK (grant C8225/A21133) and by core funding to the Clinical Trial Service Unit (from Cancer Research UK, Medical Research Council, British Heart Foundation), the British Heart Foundation Centre for Research Excellence (Grant No. RE/13/1/30181), and the UK Department of Health Policy Research Programme (Studies of Ionising Radiation and the Risk of Heart Disease, 091/0203).

## Conflict of interest statement

None of the authors disclose any financial or personal relationships with other persons or organizations that could inappropriately influence their work.
