# Supplementary material for: Risk of heart disease in relation to radiotherapy and chemotherapy with anthracyclines among 19,464 breast cancer patients in Denmark, 1977–2005
Source: Radiother Oncol. 2017 May;123(2):299–305. doi: 10.1016/j.radonc.2017.03.012 (PMC5446317; doi:10.1016/j.radonc.2017.03.012)
Supplement: Supplementary data — Supplementary Tables S1 and S2. [file mmc1.docx]

**Risk of heart disease in relation to radiotherapy and chemotherapy with anthracyclines among 19,464 breast cancer patients in Denmark, 1977-2005.**

Jens Christian Rehammar, Maj-Britt Jensen, Paul McGale, Ebbe Laugaard Lorenzen, Carolyn Taylor, Sarah C Darby, Lars Videbæk,

Zhe Wang, Marianne Ewertz

**Supplementary Tables**

|  |  |
| --- | --- |
|  |  |
| **Table S1** | **Incidence rate ratios, left-sided versus right-sided breast cancer, in women given radiotherapy by type of heart disease.** |
| **Table S2** | **Mortality rate ratios, left-sided vs right-sided, by cause of death in women given radiotherapy, according to whether or not they were also given anthracycline.** |

**Table S1. Incidence rate ratios, left-sided versus right-sided breast cancer, in women given radiotherapy by type of heart disease.**

**a) All 19,464 women for whom radiotherapy was recorded as given**

| **Disease category (ICD-10 codes)** | **Numbers of events, left/right** | **Incidence rate ratio, left vs. right***  **(95% CI)** | **p-value†** |
| --- | --- | --- | --- |
|  |  |  |  |
|  |  |  |  |
| **Ischaemic heart disease (I20-25)** | 461 / 376 | 1.18 (1.03-1.35) | 0.02 |
| Myocardial infarction (I21-23, I25.2) | 156 / 121 | 1.25 (0.98-1.59) | 0.07 |
| Angina (I20) | 218 / 170 | 1.22 (1.00-1.49) | 0.05 |
| Other ischemic heart disease | 87 / 85 | 0.97 (0.72-1.31) | 0.84 |
|  |  |  |  |
| **Other heart disease (I00-52 excluding I20-25)** | 1021 / 915 | 1.07 (0.98-1.17) | 0.13 |
| Hypertensive heart disease (I10-15) | 254 / 223 | 1.09 (0.91-1.31) | 0.33 |
| Pulmonary embolism (I26-28) | 89 / 86 | 0.98 (0.72-1.32) | 0.87 |
| Pericarditis (I01.0, I09.2, I30-32) | 47 / 36 | 1.27 (0.82-1.96) | 0.28 |
| Valvular heart disease (I00-09, I01.0, I09.2, I34-39)‡ | 82 / 58 | 1.39 (1.00-1.96) | 0.05 |
| Other rheumatic heart disease (I00.9) | 2/1 | 2.00 (0.18-22.12) | 0.57 |
| Acute endocarditis (I33) | 5/4 | 1.22 (0.33-4.54) | 0.77 |
| Myocardial disease (I40-43) | 35 / 36 | 0.96 (0.60-1.52) | 0.85 |
| Conduction disorders & arrhythmias (I44-45, I47-49) | 350 / 325 | 1.02 (0.88-1.19) | 0.77 |
| Cardiac arrest (I46) | 30 / 26 | 1.10 (0.65-1.86) | 0.72 |
| Heart failure (I50) | 110 / 106 | 0.96 (0.74-1.26) | 0.77 |
| Other & ill-defined heart disease (I51, I52) | 17 / 14 | 1.14 (0.56-2.31) | 0.73 |
|  |  |  |  |
| **All heart disease (I00-52)** | 1482 / 1291 | 1.11 (1.03-1.20) | 0.005 |

9915 women had left-sided breast cancer and 9549 women had right-sided breast cancer. Analysis based on first diagnosis of heart disease six months or more after breast cancer diagnosis.

*CI: Confidence Interval. Stratified by calendar year, age, and time since breast cancer radiotherapy.

**†**Test of whether incidence rate ratio is equal to unity.

‡Left-sided: aortic 54, mitral 19, tricuspid 2, pulmonary 2, other/unspecified 5. Right-sided: aortic 45, mitral 12, pulmonary 1.

**b) 1334 women recorded in the Danish National Patient Register as having heart disease prior to their cancer diagnosis.**

| **Disease category (ICD-10 code)** | **Number of events**  **left/right** | **Incidence rate ratio**  **left vs. right***  **(95% CI)** | **p-value^†^** |
| --- | --- | --- | --- |
|  |  |  |  |
| **Ischaemic heart disease (I20-25)** | 84/63 | 1.21 (0.87-1.70) | 0.26 |
| Myocardial infarction (I21-23, I25.2) | 25/12 | 2.05 (1.00-4.22) | 0.05 |
| Angina (I20) | 39/34 | 1.03 (0.64-1.64) | 0.91 |
| Other ischaemic heart disease | 20/17 | 0.89 (0.45-1.76) | 0.73 |
|  |  |  |  |
| **Other heart disease (I00-52 excluding I20-25)** | 143/144 | 0.96 (0.75-1.22) | 0.73 |
| Hypertensive heart disease (I10-15) | 28/41 | 0.66 (0.40-1.08) | 0.10 |
| Pulmonary embolism (I26-28) | 5/10 | 0.41 (0.13-1.26) | 0.12 |
| Pericarditis (I01.0, I09.2, I30-32) | 3/4 | 0.36 (0.06-2.04) | 0.25 |
| Valvular heart disease (I00-09, I01.0, I09.2, I34-39) | 17/14 | 1.33 (0.64-2.77) | 0.45 |
| Other rheumatic heart disease (I00.9) | 0/0 | - | - |
| Acute endocarditis (I33) | 2/1 | 1.87 (0.16-22.4) | 0.62 |
| Myocardial disease (I40-43) | 4/3 | 1.24 (0.28-5.59) | 0.78 |
| Conduction disorders & arrhythmias (I44-45, I47-49) | 69/52 | 1.26 (0.87-1.83) | 0.22 |
| Cardiac arrest (I46) | 3/5 | 0.60 (0.13-2.89) | 0.53 |
| Heart failure (I50) | 9/13 | 0.63 (0.26-1.52) | 0.30 |
| Others & ill-defined heart disease (I51, I52) | 3/1 | 3.70 (0.36-38.1) | 0.27 |
|  |  |  |  |
| **All heart disease (I00-52)** | 227/207 | 1.04 (0.86-1.28) | 0.63 |

685 women had left-sided breast cancer and 649 women had right-sided breast cancer. Analysis based on the first diagnosis of heart disease occurring six months or more after the breast cancer diagnosis.

*CI: Confidence Interval. Stratified by calendar year, age, and time since breast cancer radiotherapy.

**^†^**Test of whether incidence rate ratio, left vs right, is equal to unity.

**c) 18,130 women given radiotherapy and who were not recorded in the Danish National Patient Register as having heart disease prior to their cancer diagnosis.**

| **Disease category (ICD-10 code)** | **Number of events**  **Left/right** | **Incidence rate ratio,**  **left vs. right***  **(95% CI)** | **p-value†** |
| --- | --- | --- | --- |
|  |  |  |  |
| **Ischaemic heart disease (I20-25)** | 377/313 | 1.16 (1.00-1.35) | 0.05 |
| Myocardial infarction (I21-23, I25.2) | 131/109 | 1.18 (0.91-1.52) | 0.22 |
| Angina (I20) | 179/136 | 1.26 (1.01-1.58) | 0.04 |
| Other ischaemic heart disease | 67/68 | 0.93 (0.66-1.31) | 0.68 |
|  |  |  |  |
| **Other heart disease (I00-52 excluding I20-25)** | 878/771 | 1.10 (0.99-1.21) | 0.06 |
| Hypertensive heart disease (I10-15) | 226/182 | 1.19 (0.98-1.45) | 0.08 |
| Pulmonary embolism (I26-28) | 84/76 | 1.04 (0.76-1.42) | 0.82 |
| Pericarditis (I01.0, I09.2, I30-32) | 44/32 | 1.34 (0.85-2.12) | 0.21 |
| Valvular heart disease (I00-09, I01.0, I09.2, I34-39) | 65/44 | 1.45 (0.99-2.13) | 0.05 |
| Other rheumatic heart disease (I00.9) | 2/1 | 2.00 (0.18-22.1) | 0.57 |
| Acute endocarditis (I33) | 3/3 | 0.94 (0.18-4.65) | 0.93 |
| Myocardial disease (I40-43) | 31/33 | 0.93 (0.57-1.52) | 0.76 |
| Conduction disorders & arrhythmias (I44-45, I47-49) | 281/273 | 0.98 (0.83-1.16) | 0.84 |
| Cardiac arrest (I46) | 27/21 | 1.22 (0.69-2.17) | 0.49 |
| Heart failure (I50) | 101/93 | 1.00 (0.76-1.33) | 0.98 |
| Others & ill-defined heart disease (I51, I52) | 14/13 | 1.00 (0.47-2.13) | 0.99 |
|  |  |  |  |
| **All heart disease (I00-52)** | 1255/1084 | 1.13 (1.04-1.22) | 0.005 |

9230 women had left-sided breast cancer and 8900 women had right-sided breast cancer. Analysis based on the first diagnosis of heart disease occurring six months or more after the breast cancer diagnosis.

*CI: Confidence Interval. Stratified by calendar year, age, and time since breast cancer radiotherapy.

**†**Test of whether incidence rate ratio, left vs right, is equal to unity.

**Table S2. Mortality rate ratios, left-sided vs right-sided, by cause of death in women given radiotherapy, according to whether or not they were also given anthracycline.**

| **Cause of death (ICD-10 code)** | **Number of events**  **Left/right** | **Mortality rate ratio**  **Left vs. right***  **(95% CI)** | **p-value†** |
| --- | --- | --- | --- |
|  |  |  |  |
| **a) 3564 women given both radiotherapy and anthracycline** | | | |
| All heart disease (I00-52) | 3/9 | 0.38 (0.10-1.42) | 0.15 |
| Breast cancer (C50) | 404/337 | 1.26 (1.09-1.46) | 0.001 |
| All other causes | 72/57 | 1.39 (0.98-1.98) | 0.07 |
|  |  |  |  |
| All causes | 479/403 | 1.26 (1.10-1.44) | <0.001 |
|  |  |  |  |
| Number of women | 1777 / 1787 |  |  |
|  |  |  |  |
| **b) 15,900 women given radiotherapy but not anthracycline** | | | |
| All heart disease (I00-52) | 201/195 | 0.96 (0.79-1.17) | 0.71 |
| Breast cancer (C50) | 2419/2407 | 0.96 (0.91-1.02) | 0.19 |
| All other causes | 1042/949 | 1.05 (0.96-1.15) | 0.29 |
|  |  |  |  |
| All causes | 3662/3551 | 0.99 (0.94-1.03) | 0.55 |
|  |  |  |  |
| Number of women | 8138 / 7762 |  |  |
| **c) 19,464 women given radiotherapy** |  |  |  |
| All heart disease (I00-52) | 204 / 204 | 0.94 (0.77-1.14) | 0.51 |
| Breast cancer (C50) | 2823 / 2744 | 1.00 (0.95-1.05) | 0.91 |
| All other causes | 1114 / 1006 | 1.07 (0.98-1.16) | 0.14 |
|  |  |  |  |
| All causes | 4141 / 3954 | 1.01 (0.97-1.06) | 0.61 |
|  |  |  |  |
| Number of women | 9915 / 9549 |  |  |
|  |  |  |  |

*CI: Confidence Interval. Stratified by calendar year, age, and time since breast cancer radiotherapy.

**†**Test of whether incidence rate ratio, left vs right, is equal to unity.
